# Supplementary material for: Mechanisms governing the pioneering and redistribution capabilities of the non-classical pioneer PU.1
Source: Nat Commun. 2020 Jan 21;11:402. doi: 10.1038/s41467-019-13960-2 (PMC6972792; doi:10.1038/s41467-019-13960-2)
Supplement: Supplementary file 7 — Source data [file 41467_2019_13960_MOESM7_ESM.zip › Source_Data/FigureS4/FigureS4G_motifAnnotation/bound.singleMotif.PU1masked/knownResults.html]

/misc/data/analysis/project\_PU1/CTV1/motifs/basic/PU1.ntag15.filtered.singleMotif.PU1masked - Homer Known Motif Enrichment Results


# Homer Known Motif Enrichment Results (/misc/data/analysis/project\_PU1/CTV1/motifs/basic/PU1.ntag15.filtered.singleMotif.PU1masked)

Homer *de novo* Motif Results  
Gene Ontology Enrichment Results  
Known Motif Enrichment Results (txt file)  
Total Target Sequences = 22470, Total Background Sequences = 26532

|  |  |  |  |  |  |  |  |  |  |  |  |
| --- | --- | --- | --- | --- | --- | --- | --- | --- | --- | --- | --- |
| Rank | Motif | Name | P-value | log P-pvalue | q-value (Benjamini) | # Target Sequences with Motif | % of Targets Sequences with Motif | # Background Sequences with Motif | % of Background Sequences with Motif | Motif File | SVG |
| 1 | T C G A T A G C T G C A A C T G A C T G C G T A C G T A C T A G G A C T T A C G | ETS1(ETS)/Jurkat-ETS1-ChIP-Seq(GSE17954)/Homer | 1e-133 | -3.063e+02 | 0.0000 | 2620.0 | 11.66% | 1461.9 | 5.51% | motif file (matrix) | svg |
| 2 | T C G A C T G A T A G C T G A C T C A G T C A G C G T A C G T A T C A G A G C T | ETV1(ETS)/GIST48-ETV1-ChIP-Seq(GSE22441)/Homer | 1e-120 | -2.783e+02 | 0.0000 | 3815.0 | 16.97% | 2602.7 | 9.80% | motif file (matrix) | svg |
| 3 | T C G A T C G A T A G C G T A C T C A G T A C G C G T A C G T A T C A G A G C T | GABPA(ETS)/Jurkat-GABPa-ChIP-Seq(GSE17954)/Homer | 1e-98 | -2.280e+02 | 0.0000 | 2427.0 | 10.80% | 1485.5 | 5.60% | motif file (matrix) | svg |
| 4 | T C G A T A G C G T C A A C T G A C T G C G T A C G T A C T A G A G C T T C A G | ERG(ETS)/VCaP-ERG-ChIP-Seq(GSE14097)/Homer | 1e-98 | -2.274e+02 | 0.0000 | 4601.0 | 20.47% | 3537.6 | 13.33% | motif file (matrix) | svg |
| 5 | A T G C A G T C C T G A A G T C C G A T A C G T A G T C A G T C A C G T A T C G G A C T A C G T | Etv2(ETS)/ES-ER71-ChIP-Seq(GSE59402)/Homer(0.967) | 1e-96 | -2.232e+02 | 0.0000 | 2636.0 | 11.73% | 1683.8 | 6.34% | motif file (matrix) | svg |
| 6 | A G T C C T G A A G T C C G A T C A G T G A T C A T G C A C T G A T C G G A C T | Fli1(ETS)/CD8-FLI-ChIP-Seq(GSE20898)/Homer | 1e-94 | -2.167e+02 | 0.0000 | 2790.0 | 12.41% | 1841.2 | 6.94% | motif file (matrix) | svg |
| 7 | G C T A C T G A T C G A A G T C A G T C C T G A A G T C G T C A C T G A T G C A | RUNX1(Runt)/Jurkat-RUNX1-ChIP-Seq(GSE29180)/Homer | 1e-85 | -1.974e+02 | 0.0000 | 3499.0 | 15.57% | 2570.5 | 9.68% | motif file (matrix) | svg |
| 8 | T G C A C T G A A T G C G T C A A C T G A C T G C G T A C G T A C T A G A G C T | Ets1-distal(ETS)/CD4+-PolII-ChIP-Seq(Barski\_et\_al.)/Homer | 1e-78 | -1.818e+02 | 0.0000 | 954.0 | 4.24% | 390.4 | 1.47% | motif file (matrix) | svg |
| 9 | T A G C G C T A T C G A C T G A A G T C A G T C C T G A A G T C C G T A C T A G | RUNX(Runt)/HPC7-Runx1-ChIP-Seq(GSE22178)/Homer | 1e-78 | -1.798e+02 | 0.0000 | 2481.0 | 11.04% | 1671.1 | 6.30% | motif file (matrix) | svg |
| 10 | C G T A T A G C T A G C T G C A A C T G C T A G C G T A C G T A T C A G G A C T | EHF(ETS)/LoVo-EHF-ChIP-Seq(GSE49402)/Homer | 1e-71 | -1.638e+02 | 0.0000 | 3270.0 | 14.55% | 2474.6 | 9.32% | motif file (matrix) | svg |
| 11 | T G C A A G C T C T G A A T C G G A C T C T A G G T A C G A T C G T C A A G T C G T A C G A C T C T A G A T C G G C A T C A T G C A T G G A T C G T A C C T G A | CTCF(Zf)/CD4+-CTCF-ChIP-Seq(Barski\_et\_al.)/Homer | 1e-70 | -1.615e+02 | 0.0000 | 550.0 | 2.45% | 151.1 | 0.57% | motif file (matrix) | svg |
| 12 | T G C A C T G A A G T C G T C A A C T G A C T G C G T A C G T A C T G A A G C T | EWS:FLI1-fusion(ETS)/SK\_N\_MC-EWS:FLI1-ChIP-Seq(SRA014231)/Homer | 1e-69 | -1.596e+02 | 0.0000 | 1825.0 | 8.12% | 1141.9 | 4.30% | motif file (matrix) | svg |
| 13 | T G A C G C T A T C G A T G C A A G T C A G T C C G T A A G T C C G T A C T G A G C T A G T A C | RUNX2(Runt)/PCa-RUNX2-ChIP-Seq(GSE33889)/Homer | 1e-60 | -1.401e+02 | 0.0000 | 2753.0 | 12.25% | 2065.8 | 7.78% | motif file (matrix) | svg |
| 14 | T C G A A G C T A C G T A C G T A G T C A G T C A C G T A T C G G A C T A T C G | EWS:ERG-fusion(ETS)/CADO\_ES1-EWS:ERG-ChIP-Seq(SRA014231)/Homer | 1e-60 | -1.396e+02 | 0.0000 | 2134.0 | 9.49% | 1481.2 | 5.58% | motif file (matrix) | svg |
| 15 | A C T G G A T C G A C T A C T G A C G T C A T G A C T G A C G T A G C T C G A T | RUNX-AML(Runt)/CD4+-PolII-ChIP-Seq(Barski\_et\_al.)/Homer | 1e-57 | -1.318e+02 | 0.0000 | 2412.0 | 10.73% | 1770.8 | 6.67% | motif file (matrix) | svg |
| 16 | G C T A A G T C T A C G T G C A A T C G T C A G G C T A T C G A T C A G A G C T | ELF5(ETS)/T47D-ELF5-ChIP-Seq(GSE30407)/Homer | 1e-55 | -1.276e+02 | 0.0000 | 1394.0 | 6.20% | 853.6 | 3.22% | motif file (matrix) | svg |
| 17 | G A T C T C G A A G T C C G A T C G A T A G T C A T G C A C T G A T C G G A C T | Elk1(ETS)/Hela-Elk1-ChIP-Seq(GSE31477)/Homer | 1e-54 | -1.251e+02 | 0.0000 | 1218.0 | 5.42% | 709.3 | 2.67% | motif file (matrix) | svg |
| 18 | T A G C A G T C T G A C A G T C C T A G A T C G A G T C C A T G T G A C A G T C G T A C A G T C A G T C G C A T C T A G A T C G G C A T A C T G A T C G G A T C | BORIS(Zf)/K562-CTCFL-ChIP-Seq(GSE32465)/Homer | 1e-51 | -1.193e+02 | 0.0000 | 598.0 | 2.66% | 236.5 | 0.89% | motif file (matrix) | svg |
| 19 | C G T A T G A C T A G C T G C A A C T G A C T G C G T A C G T A T C A G G A C T | ELF3(ETS)/PDAC-ELF3-ChIP-Seq(GSE64557)/Homer | 1e-48 | -1.127e+02 | 0.0000 | 1662.0 | 7.39% | 1136.2 | 4.28% | motif file (matrix) | svg |
| 20 | G A T C C T G A A G T C C G A T C G A T G A T C A G T C A C T G A T C G A G C T | Elk4(ETS)/Hela-Elk4-ChIP-Seq(GSE31477)/Homer | 1e-45 | -1.052e+02 | 0.0000 | 1225.0 | 5.45% | 768.5 | 2.89% | motif file (matrix) | svg |
| 21 | C T G A A T G C C G T A A C G T A G T C A G T C A C G T A C T G A T C G G C A T | SPDEF(ETS)/VCaP-SPDEF-ChIP-Seq(SRA014231)/Homer | 1e-44 | -1.030e+02 | 0.0000 | 2625.0 | 11.68% | 2096.0 | 7.90% | motif file (matrix) | svg |
| 22 | T G C A T C G A T A G C G T A C T C A G C T A G G T C A G C T A T C A G G A C T | ETS(ETS)/Promoter/Homer | 1e-43 | -9.999e+01 | 0.0000 | 694.0 | 3.09% | 340.4 | 1.28% | motif file (matrix) | svg |
| 23 | C T G A T G C A T A G C T G A C T A C G T C A G C T G A G C T A T C A G G A C T | ELF1(ETS)/Jurkat-ELF1-ChIP-Seq(SRA014231)/Homer | 1e-41 | -9.584e+01 | 0.0000 | 1036.0 | 4.61% | 630.9 | 2.38% | motif file (matrix) | svg |
| 24 | C A T G C T A G T C G A A C G T A C T G C G T A T A G C C G A T T G A C C G T A A G C T G A T C | Fra2(bZIP)/Striatum-Fra2-ChIP-Seq(GSE43429)/Homer | 1e-32 | -7.537e+01 | 0.0000 | 1354.0 | 6.02% | 982.0 | 3.70% | motif file (matrix) | svg |
| 25 | C T A G T C G A C G A T A C T G C G T A T A C G A G C T T G A C G C T A A C G T G A T C T A G C | Fosl2(bZIP)/3T3L1-Fosl2-ChIP-Seq(GSE56872)/Homer | 1e-32 | -7.413e+01 | 0.0000 | 977.0 | 4.35% | 641.7 | 2.42% | motif file (matrix) | svg |
| 26 | A C T G C T A G T C G A C G A T C A T G G C T A A T C G C G A T G T A C G C T A A G C T G T A C | Fra1(bZIP)/BT549-Fra1-ChIP-Seq(GSE46166)/Homer | 1e-31 | -7.201e+01 | 0.0000 | 1540.0 | 6.85% | 1172.4 | 4.42% | motif file (matrix) | svg |
| 27 | C T A G T C G A G C A T C A T G G C T A T A G C C G A T G T A C C T G A A G C T | JunB(bZIP)/DendriticCells-Junb-ChIP-Seq(GSE36099)/Homer | 1e-30 | -6.925e+01 | 0.0000 | 1560.0 | 6.94% | 1203.2 | 4.53% | motif file (matrix) | svg |
| 28 | C T A G T C G A A C G T A C T G C G T A T A G C C G A T G T A C C G T A A G C T G A T C G T A C | Jun-AP1(bZIP)/K562-cJun-ChIP-Seq(GSE31477)/Homer | 1e-29 | -6.786e+01 | 0.0000 | 739.0 | 3.29% | 451.2 | 1.70% | motif file (matrix) | svg |
| 29 | C T G A T C A G G T A C G C T A A C T G T G A C G C A T C A T G | SCL(bHLH)/HPC7-Scl-ChIP-Seq(GSE13511)/Homer | 1e-27 | -6.397e+01 | 0.0000 | 10763.0 | 47.88% | 11391.5 | 42.91% | motif file (matrix) | svg |
| 30 | C T A G T C G A A C G T A C T G C G T A A T G C A C G T G T A C C G T A A G C T G A T C G T A C | Atf3(bZIP)/GBM-ATF3-ChIP-Seq(GSE33912)/Homer | 1e-27 | -6.350e+01 | 0.0000 | 1826.0 | 8.12% | 1491.2 | 5.62% | motif file (matrix) | svg |
| 31 | T C G A T A G C G T C A A C T G C T A G C G T A C G A T A C T G A C G T A C T G A C T G A C G T | ETS:RUNX(ETS,Runt)/Jurkat-RUNX1-ChIP-Seq(GSE17954)/Homer | 1e-27 | -6.223e+01 | 0.0000 | 290.0 | 1.29% | 109.2 | 0.41% | motif file (matrix) | svg |
| 32 | T C G A A C G T C A T G G C T A T A G C C G A T G T A C G C T A A C G T A T G C | AP-1(bZIP)/ThioMac-PU.1-ChIP-Seq(GSE21512)/Homer | 1e-26 | -6.184e+01 | 0.0000 | 2000.0 | 8.90% | 1674.1 | 6.31% | motif file (matrix) | svg |
| 33 | C A G T T G C A A C G T A C T G C G T A A T C G C G A T T G A C C G T A A C G T | BATF(bZIP)/Th17-BATF-ChIP-Seq(GSE39756)/Homer | 1e-25 | -5.958e+01 | 0.0000 | 1786.0 | 7.95% | 1471.6 | 5.54% | motif file (matrix) | svg |
| 34 | C G A T T A C G T G A C G A C T C A T G C G T A T A C G A C G T G T A C C T G A | Bach2(bZIP)/OCILy7-Bach2-ChIP-Seq(GSE44420)/Homer | 1e-20 | -4.650e+01 | 0.0000 | 565.0 | 2.51% | 361.4 | 1.36% | motif file (matrix) | svg |
| 35 | C G T A C G T A C G T A G C A T G C A T T A C G G T A C G A C T A C T G C G T A A T C G A C G T G T A C C G T A A G C T | Bach1(bZIP)/K562-Bach1-ChIP-Seq(GSE31477)/Homer | 1e-18 | -4.232e+01 | 0.0000 | 206.0 | 0.92% | 81.5 | 0.31% | motif file (matrix) | svg |
| 36 | C G T A C T G A C G T A C T A G T C G A C T A G A C T G C G T A C G T A T A C G A G C T A T C G | SpiB(ETS)/OCILY3-SPIB-ChIP-Seq(GSE56857)/Homer | 1e-18 | -4.205e+01 | 0.0000 | 106.0 | 0.47% | 20.5 | 0.08% | motif file (matrix) | svg |
| 37 | G T C A G C A T A C T G G T A C G A C T A C T G G C T A A T C G C A G T G T A C C G T A A G C T | Nrf2(bZIP)/Lymphoblast-Nrf2-ChIP-Seq(GSE37589)/Homer | 1e-14 | -3.436e+01 | 0.0000 | 181.0 | 0.81% | 76.3 | 0.29% | motif file (matrix) | svg |
| 38 | C T A G C T A G C G T A C G T A T A C G C G A T C T A G C T G A C T G A C G T A T A C G G A C T | PU.1:IRF8(ETS:IRF)/pDC-Irf8-ChIP-Seq(GSE66899)/Homer | 1e-14 | -3.267e+01 | 0.0000 | 528.0 | 2.35% | 373.2 | 1.41% | motif file (matrix) | svg |
| 39 | T A C G T C G A C A G T A C T G G C T A A T G C C G A T G T A C C G T A A C T G T A G C C G T A | NF-E2(bZIP)/K562-NFE2-ChIP-Seq(GSE31477)/Homer | 1e-13 | -3.180e+01 | 0.0000 | 202.0 | 0.90% | 96.9 | 0.37% | motif file (matrix) | svg |
| 40 | G A C T C T A G C T A G C T A G A C T G T C G A C T G A C T A G C T A G C T A G G T A C G T C A | ZNF467(Zf)/HEK293-ZNF467.GFP-ChIP-Seq(GSE58341)/Homer | 1e-13 | -3.126e+01 | 0.0000 | 2158.0 | 9.60% | 2040.9 | 7.69% | motif file (matrix) | svg |
| 41 | C T A G C T A G A G T C T C A G A C T G A C G T A C G T C T G A | MYB(HTH)/ERMYB-Myb-ChIPSeq(GSE22095)/Homer | 1e-13 | -3.079e+01 | 0.0000 | 3886.0 | 17.29% | 3930.6 | 14.81% | motif file (matrix) | svg |
| 42 | C G T A T A C G T C G A A C T G A C T G C G T A C G T A T A C G A G C T T A C G | PU.1(ETS)/ThioMac-PU.1-ChIP-Seq(GSE21512)/Homer | 1e-12 | -2.932e+01 | 0.0000 | 280.0 | 1.25% | 164.5 | 0.62% | motif file (matrix) | svg |
| 43 | T C A G C G T A A G T C A G C T C G T A A G T C C T G A C G T A A G T C G C A T A G T C A G T C A G T C C T G A A C T G T G C A T C G A C A T G A T C G G A T C | Ronin(THAP)/ES-Thap11-ChIP-Seq(GSE51522)/Homer | 1e-12 | -2.831e+01 | 0.0000 | 103.0 | 0.46% | 32.9 | 0.12% | motif file (matrix) | svg |
| 44 | A G T C G C A T C G T A C G T A G T A C A C G T A C T G G A T C G A T C T C G A | BMYB(HTH)/Hela-BMYB-ChIP-Seq(GSE27030)/Homer | 1e-12 | -2.779e+01 | 0.0000 | 3422.0 | 15.22% | 3450.9 | 13.00% | motif file (matrix) | svg |
| 45 | G T C A T G C A G C T A A G T C C G T A A C T G T G A C G C A T T C A G C A G T | Ap4(bHLH)/AML-Tfap4-ChIP-Seq(GSE45738)/Homer | 1e-11 | -2.674e+01 | 0.0000 | 2715.0 | 12.08% | 2684.1 | 10.11% | motif file (matrix) | svg |
| 46 | A G C T A G T C A T G C A G C T A C G T C G T A A C G T A G T C C G A T A T G C | Gata2(Zf)/K562-GATA2-ChIP-Seq(GSE18829)/Homer | 1e-10 | -2.504e+01 | 0.0000 | 1718.0 | 7.64% | 1623.7 | 6.12% | motif file (matrix) | svg |
| 47 | A G C T A T G C G A C T G C A T C G T A A G C T G T A C C G A T A T C G A G T C | Gata6(Zf)/HUG1N-GATA6-ChIP-Seq(GSE51936)/Homer | 1e-10 | -2.476e+01 | 0.0000 | 2335.0 | 10.39% | 2290.9 | 8.63% | motif file (matrix) | svg |
| 48 | C T A G A C T G C T A G T C A G T C A G T A C G C T A G A C T G | Maz(Zf)/HepG2-Maz-ChIP-Seq(GSE31477)/Homer | 1e-10 | -2.392e+01 | 0.0000 | 2715.0 | 12.08% | 2714.5 | 10.23% | motif file (matrix) | svg |
| 49 | C G A T T G C A T G C A G A T C C G T A A C T G T G A C G A C T C A T G A C T G | Tcf21(bHLH)/ArterySmoothMuscle-Tcf21-ChIP-Seq(GSE61369)/Homer | 1e-10 | -2.367e+01 | 0.0000 | 2102.0 | 9.35% | 2049.0 | 7.72% | motif file (matrix) | svg |
| 50 | T A C G A T G C G C T A A C T G C G T A A C G T C G T A C T G A T A C G T C G A | Gata4(Zf)/Heart-Gata4-ChIP-Seq(GSE35151)/Homer | 1e-10 | -2.355e+01 | 0.0000 | 2591.0 | 11.53% | 2582.2 | 9.73% | motif file (matrix) | svg |
| 51 | T A G C G C T A A C T G C G T A A C G T C G T A C G T A T A C G T C A G T C G A | Gata1(Zf)/K562-GATA1-ChIP-Seq(GSE18829)/Homer | 1e-10 | -2.347e+01 | 0.0000 | 1525.0 | 6.78% | 1431.5 | 5.39% | motif file (matrix) | svg |
| 52 | T C G A A G T C C G T A A T C G A T G C C G A T A C T G A G T C A G C T A C T G | Tcf12(bHLH)/GM12878-Tcf12-ChIP-Seq(GSE32465)/Homer | 1e-10 | -2.344e+01 | 0.0000 | 2135.0 | 9.50% | 2086.3 | 7.86% | motif file (matrix) | svg |
| 53 | A C G T T C G A T C G A A G T C G T C A T A C G A T G C A C G T A C T G A G C T | Myf5(bHLH)/GM-Myf5-ChIP-Seq(GSE24852)/Homer | 1e-10 | -2.339e+01 | 0.0000 | 1547.0 | 6.88% | 1455.7 | 5.48% | motif file (matrix) | svg |
| 54 | T A C G T C A G A G C T A T G C C G T A A G T C T C A G A C G T A C T G T C G A | USF1(bHLH)/GM12878-Usf1-ChIP-Seq(GSE32465)/Homer | 1e-9 | -2.289e+01 | 0.0000 | 1004.0 | 4.47% | 890.5 | 3.35% | motif file (matrix) | svg |
| 55 | T G A C G C T A T G A C C G T A T C A G G A T C C G T A C A T G C A T G C T A G C T A G C T A G | Unknown-ESC-element(?)/mES-Nanog-ChIP-Seq(GSE11724)/Homer | 1e-9 | -2.238e+01 | 0.0000 | 1475.0 | 6.56% | 1387.8 | 5.23% | motif file (matrix) | svg |
| 56 | T C A G A G C T A T G C C G T A A G C T T C A G C A G T A C T G C T G A A G T C | MITF(bHLH)/MastCells-MITF-ChIP-Seq(GSE48085)/Homer | 1e-9 | -2.163e+01 | 0.0000 | 2121.0 | 9.44% | 2089.8 | 7.87% | motif file (matrix) | svg |
| 57 | C T G A T C A G A G T C C G T A A T C G A T G C C G A T A C T G A G T C G A C T A T C G A G T C | MyoD(bHLH)/Myotube-MyoD-ChIP-Seq(GSE21614)/Homer | 1e-8 | -2.030e+01 | 0.0000 | 1591.0 | 7.08% | 1529.5 | 5.76% | motif file (matrix) | svg |
| 58 | C A G T T C A G G A T C A C T G A C G T C T A G A C T G A C T G G A C T C T A G | Egr1(Zf)/K562-Egr1-ChIP-Seq(GSE32465)/Homer | 1e-8 | -2.029e+01 | 0.0000 | 1288.0 | 5.73% | 1206.1 | 4.54% | motif file (matrix) | svg |
| 59 | C A T G T A C G T A G C G A T C G A T C A T G C G T A C G A C T T C A G A T G C C G A T A T C G C A G T A C T G G T A C | Zic3(Zf)/mES-Zic3-ChIP-Seq(GSE37889)/Homer | 1e-8 | -1.972e+01 | 0.0000 | 1276.0 | 5.68% | 1198.6 | 4.52% | motif file (matrix) | svg |
| 60 | C G T A A T G C C G A T A C G T A G T C C G T A C G T A C G T A C T A G A T C G | TCFL2(HMG)/K562-TCF7L2-ChIP-Seq(GSE29196)/Homer | 1e-8 | -1.868e+01 | 0.0000 | 238.0 | 1.06% | 158.7 | 0.60% | motif file (matrix) | svg |
| 61 | G A C T C T A G G A T C C A G T A C T G C T G A A T G C G C A T A T G C C T G A | MafA(bZIP)/Islet-MafA-ChIP-Seq(GSE30298)/Homer | 1e-8 | -1.844e+01 | 0.0000 | 1695.0 | 7.54% | 1659.9 | 6.25% | motif file (matrix) | svg |
| 62 | A G T C A T C G C T A G A G C T G A C T C T A G A G T C A G T C G C T A C A G T T C A G T C A G G A T C C T G A T C G A G A T C | RFX(HTH)/K562-RFX3-ChIP-Seq(SRA012198)/Homer | 1e-7 | -1.793e+01 | 0.0000 | 177.0 | 0.79% | 107.1 | 0.40% | motif file (matrix) | svg |
| 63 | A G T C A C G T A C T G A G C T A C G T A C G T G T C A A G T C | Foxo1(Forkhead)/RAW-Foxo1-ChIP-Seq(Fan\_et\_al.)/Homer | 1e-7 | -1.790e+01 | 0.0000 | 4827.0 | 21.48% | 5164.2 | 19.45% | motif file (matrix) | svg |
| 64 | T C G A C G T A A G T C A G C T C G T A A G T C T C G A G C T A G A C T C G A T A G T C A G T C A G T C C T G A T C A G T G C A T C G A C A G T A T C G A G T C | GFY-Staf(?,Zf)/Promoter/Homer | 1e-7 | -1.785e+01 | 0.0000 | 137.0 | 0.61% | 74.7 | 0.28% | motif file (matrix) | svg |
| 65 | C T G A A T G C G C T A G C A T A T G C C G T A T C G A C T G A C T A G T C A G T A C G G T C A | Tcf4(HMG)/Hct116-Tcf4-ChIP-Seq(SRA012054)/Homer | 1e-7 | -1.749e+01 | 0.0000 | 1242.0 | 5.53% | 1182.0 | 4.45% | motif file (matrix) | svg |
| 66 | T C G A T C G A A G T C C G T A C T A G T A G C A C G T A C T G | MyoG(bHLH)/C2C12-MyoG-ChIP-Seq(GSE36024)/Homer | 1e-7 | -1.732e+01 | 0.0000 | 2278.0 | 10.13% | 2310.0 | 8.70% | motif file (matrix) | svg |
| 67 | G C T A A T C G G C T A G A C T G C T A T C G A T A G C T C G A | GATA3(Zf)/iTreg-Gata3-ChIP-Seq(GSE20898)/Homer | 1e-7 | -1.717e+01 | 0.0000 | 3813.0 | 16.96% | 4026.2 | 15.17% | motif file (matrix) | svg |
| 68 | G A C T C T A G C T A G A G T C T G C A A C T G A C G T A C G T C T A G T C A G | AMYB(HTH)/Testes-AMYB-ChIP-Seq(GSE44588)/Homer | 1e-7 | -1.661e+01 | 0.0000 | 3390.0 | 15.08% | 3558.0 | 13.40% | motif file (matrix) | svg |
| 69 | C T G A A T G C G C T A C G A T A T G C C G T A C G T A C G T A C T A G T A C G | Tcf3(HMG)/mES-Tcf3-ChIP-Seq(GSE11724)/Homer | 1e-7 | -1.616e+01 | 0.0000 | 692.0 | 3.08% | 614.6 | 2.32% | motif file (matrix) | svg |
| 70 | T C A G A C G T T C G A T A G C A G T C C G T A A C T G G T A C A C G T A C T G A T C G A G T C | Atoh1(bHLH)/Cerebellum-Atoh1-ChIP-Seq(GSE22111)/Homer | 1e-6 | -1.597e+01 | 0.0000 | 2428.0 | 10.80% | 2492.8 | 9.39% | motif file (matrix) | svg |
| 71 | C T G A A T C G A G C T A G C T A C G T T A G C C T G A T A C G C G A T A C G T G A C T A G T C | ISRE(IRF)/ThioMac-LPS-Expression(GSE23622)/Homer | 1e-6 | -1.537e+01 | 0.0000 | 218.0 | 0.97% | 151.8 | 0.57% | motif file (matrix) | svg |
| 72 | C T G A T A C G G C A T A G C T A G C T A G T C T C G A A C T G C A G T A G C T A G C T G A T C | IRF3(IRF)/BMDM-Irf3-ChIP-Seq(GSE67343)/Homer | 1e-6 | -1.514e+01 | 0.0000 | 877.0 | 3.90% | 814.0 | 3.07% | motif file (matrix) | svg |
| 73 | C T A G A G C T G A C T C A T G A G T C A G T C G T C A C A G T C T A G T C A G G T A C C T G A T C G A G A T C T G A C | Rfx2(HTH)/LoVo-RFX2-ChIP-Seq(GSE49402)/Homer | 1e-6 | -1.511e+01 | 0.0000 | 196.0 | 0.87% | 132.8 | 0.50% | motif file (matrix) | svg |
| 74 | T C A G T G A C G T A C C G T A A C G T T G A C A C G T T C A G A G C T G A C T | NeuroD1(bHLH)/Islet-NeuroD1-ChIP-Seq(GSE30298)/Homer | 1e-6 | -1.442e+01 | 0.0000 | 1874.0 | 8.34% | 1899.3 | 7.15% | motif file (matrix) | svg |
| 75 | T G C A T A G C G A C T T G C A T G A C T G C A C G T A A G C T A G C T A G T C A G T C G T A C | GFY(?)/Promoter/Homer | 1e-6 | -1.415e+01 | 0.0000 | 188.0 | 0.84% | 128.2 | 0.48% | motif file (matrix) | svg |
| 76 | C T A G T C G A C T G A C G T A T A C G G A C T T C A G T C G A G T C A T G C A T A C G A G C T | IRF2(IRF)/Erythroblas-IRF2-ChIP-Seq(GSE36985)/Homer | 1e-5 | -1.360e+01 | 0.0000 | 306.0 | 1.36% | 241.0 | 0.91% | motif file (matrix) | svg |
| 77 | C T A G C A G T T G A C C G T A G A T C T C A G G A C T C A T G | BMAL1(bHLH)/Liver-Bmal1-ChIP-Seq(GSE39860)/Homer | 1e-5 | -1.316e+01 | 0.0000 | 4039.0 | 17.97% | 4350.7 | 16.39% | motif file (matrix) | svg |
| 78 | T C A G A G C T A T G C C G T A A G T C T C A G A C G T A T C G T C G A A G T C G A T C T G A C | TFE3(bHLH)/MEF-TFE3-ChIP-Seq(GSE75757)/Homer | 1e-5 | -1.254e+01 | 0.0000 | 196.0 | 0.87% | 141.6 | 0.53% | motif file (matrix) | svg |
| 79 | T C A G T A G C G A C T C A T G C T G A A T C G G C A T G T A C C G T A A C T G T A G C T G C A | MafK(bZIP)/C2C12-MafK-ChIP-Seq(GSE36030)/Homer | 1e-5 | -1.175e+01 | 0.0000 | 493.0 | 2.19% | 439.1 | 1.65% | motif file (matrix) | svg |
| 80 | T G A C C T A G T C A G G T C A C G T A T C A G C G A T T C A G T C G A T G C A C T G A T A G C | PU.1-IRF(ETS:IRF)/Bcell-PU.1-ChIP-Seq(GSE21512)/Homer | 1e-4 | -1.101e+01 | 0.0001 | 3595.0 | 15.99% | 3885.6 | 14.64% | motif file (matrix) | svg |
| 81 | C T A G T C G A C G A T C T A G G C A T C A G T C T A G G A T C C G T A G T C A | CEBP:AP1(bZIP)/ThioMac-CEBPb-ChIP-Seq(GSE21512)/Homer | 1e-4 | -1.091e+01 | 0.0001 | 1745.0 | 7.76% | 1802.9 | 6.79% | motif file (matrix) | svg |
| 82 | T C A G T C A G G C T A C G T A T A C G G A C T T C A G T C G A C T G A C G T A T A C G G A C T | IRF8(IRF)/BMDM-IRF8-ChIP-Seq(GSE77884)/Homer | 1e-4 | -1.088e+01 | 0.0001 | 844.0 | 3.75% | 816.4 | 3.08% | motif file (matrix) | svg |
| 83 | C A T G C T A G A G C T G A C T C A T G A G T C G A T C G C T A C G A T C T A G T C A G G T A C C T G A T C G A | X-box(HTH)/NPC-H3K4me1-ChIP-Seq(GSE16256)/Homer | 1e-4 | -1.082e+01 | 0.0001 | 240.0 | 1.07% | 190.4 | 0.72% | motif file (matrix) | svg |
| 84 | T C G A T G A C A G T C C G T A A G T C C T A G A C G T A C T G A C T G A G C T A G T C G C A T | Max(bHLH)/K562-Max-ChIP-Seq(GSE31477)/Homer | 1e-4 | -1.080e+01 | 0.0001 | 1339.0 | 5.96% | 1355.3 | 5.11% | motif file (matrix) | svg |
| 85 | T C G A T G A C G T A C C G T A C A G T T G A C A C G T A C T G A G C T A G C T | NeuroG2(bHLH)/Fibroblast-NeuroG2-ChIP-Seq(GSE75910)/Homer | 1e-4 | -1.013e+01 | 0.0002 | 3520.0 | 15.66% | 3817.5 | 14.38% | motif file (matrix) | svg |
| 86 | C T G A T C A G C A G T C T A G A C T G C T A G G A T C A T C G A C T G C T G A T C A G G A T C | Sp5(Zf)/mES-Sp5.Flag-ChIP-Seq(GSE72989)/Homer | 1e-4 | -9.880e+00 | 0.0002 | 1977.0 | 8.80% | 2076.8 | 7.82% | motif file (matrix) | svg |
| 87 | A C T G T C A G A G C T G A C T C A T G A G T C A G T C G C T A C G A T C T A G T C A G G T A C C T G A T C G A | Rfx1(HTH)/NPC-H3K4me1-ChIP-Seq(GSE16256)/Homer | 1e-4 | -9.590e+00 | 0.0003 | 433.0 | 1.93% | 392.6 | 1.48% | motif file (matrix) | svg |
| 88 | T C G A C T A G A G T C A G T C C G T A C G T A A C G T T A G C T C A G T A C G | NFY(CCAAT)/Promoter/Homer | 1e-4 | -9.457e+00 | 0.0003 | 1551.0 | 6.90% | 1607.6 | 6.06% | motif file (matrix) | svg |
| 89 | T C A G A C G T A G T C T C G A A G T C T C A G G C A T C T A G C T A G A G C T | Usf2(bHLH)/C2C12-Usf2-ChIP-Seq(GSE36030)/Homer | 1e-4 | -9.453e+00 | 0.0003 | 739.0 | 3.29% | 718.0 | 2.70% | motif file (matrix) | svg |
| 90 | C G T A G A C T C G A T A T C G G T A C G C A T C A T G C G T A T A C G G C A T G T A C C G T A C A T G A T G C G C T A C T A G G C A T G C A T G C A T G A C T | MafB(bZIP)/BMM-Mafb-ChIP-Seq(GSE75722)/Homer | 1e-4 | -9.356e+00 | 0.0003 | 889.0 | 3.96% | 880.0 | 3.32% | motif file (matrix) | svg |
| 91 | A G C T A C G T A C T G A T G C A G T C C G T A C T G A T A C G | NF1-halfsite(CTF)/LNCaP-NF1-ChIP-Seq(Unpublished)/Homer | 1e-3 | -8.862e+00 | 0.0006 | 3962.0 | 17.63% | 4350.8 | 16.39% | motif file (matrix) | svg |
| 92 | C T A G A G T C T A C G T A C G T G A C C G T A A C T G T A G C G C A T C A T G A T G C A G C T | Ascl1(bHLH)/NeuralTubes-Ascl1-ChIP-Seq(GSE55840)/Homer | 1e-3 | -8.209e+00 | 0.0011 | 3387.0 | 15.07% | 3706.3 | 13.96% | motif file (matrix) | svg |
| 93 | A G C T C A T G G C A T G A T C T G C A C T A G G A T C A C G T | Tgif2(Homeobox)/mES-Tgif2-ChIP-Seq(GSE55404)/Homer | 1e-3 | -7.889e+00 | 0.0015 | 7690.0 | 34.21% | 8698.2 | 32.77% | motif file (matrix) | svg |
| 94 | T C G A A C T G A C T G C G T A C G T A T C G A A G T C C T G A A T C G G T A C G C A T C A T G | ETS:E-box(ETS,bHLH)/HPC7-Scl-ChIP-Seq(GSE22178)/Homer | 1e-3 | -7.844e+00 | 0.0015 | 273.0 | 1.21% | 239.7 | 0.90% | motif file (matrix) | svg |
| 95 | A C G T T A C G G A T C A C T G A C G T C T A G A C T G A C T G G A T C C T A G C A T G C T A G | Egr2(Zf)/Thymocytes-Egr2-ChIP-Seq(GSE34254)/Homer | 1e-3 | -7.838e+00 | 0.0015 | 271.0 | 1.21% | 237.6 | 0.89% | motif file (matrix) | svg |
| 96 | T C G A G C A T A C G T C T A G G T A C T C G A G C A T T G A C T C G A A C G T | Chop(bZIP)/MEF-Chop-ChIP-Seq(GSE35681)/Homer | 1e-3 | -7.698e+00 | 0.0017 | 521.0 | 2.32% | 500.5 | 1.89% | motif file (matrix) | svg |
| 97 | T A C G T C A G T G C A A G C T T G A C A G C T A G T C A C T G G A T C A C T G T C G A A C T G C T G A C T G A A T G C | ZBTB33(Zf)/GM12878-ZBTB33-ChIP-Seq(GSE32465)/Homer | 1e-3 | -7.099e+00 | 0.0031 | 55.0 | 0.24% | 32.5 | 0.12% | motif file (matrix) | svg |
| 98 | C T G A T G A C T G A C C G T A A C G T T G A C A G C T C T A G A C G T G A C T | Olig2(bHLH)/Neuron-Olig2-ChIP-Seq(GSE30882)/Homer | 1e-3 | -7.024e+00 | 0.0033 | 4443.0 | 19.77% | 4950.3 | 18.65% | motif file (matrix) | svg |
| 99 | A T G C A T C G T A C G A G C T A T C G C T G A A G T C C T A G A G C T A T G C C T G A A T G C | CRE(bZIP)/Promoter/Homer | 1e-3 | -6.981e+00 | 0.0034 | 353.0 | 1.57% | 328.1 | 1.24% | motif file (matrix) | svg |
| 100 | T A C G A T G C G A C T A C T G A G C T A G T C G T C A T G C A A C G T A G T C G C T A T G C A | Pknox1(Homeobox)/ES-Prep1-ChIP-Seq(GSE63282)/Homer | 1e-3 | -6.965e+00 | 0.0034 | 452.0 | 2.01% | 433.8 | 1.63% | motif file (matrix) | svg |
| 101 | T G C A A G C T C A T G C G T A A G C T A C T G G A T C G T C A C G T A A G C T | Atf4(bZIP)/MEF-Atf4-ChIP-Seq(GSE35681)/Homer | 1e-2 | -6.799e+00 | 0.0040 | 636.0 | 2.83% | 633.7 | 2.39% | motif file (matrix) | svg |
| 102 | A T G C C G T A A C T G C G T A A C G T G C T A T C G A A G C T C G A T C G T A A C G T A G T C C G A T A C T G G A T C | GATA(Zf),IR4/iTreg-Gata3-ChIP-Seq(GSE20898)/Homer | 1e-2 | -6.637e+00 | 0.0047 | 183.0 | 0.81% | 155.1 | 0.58% | motif file (matrix) | svg |
| 103 | A T G C A T G C A T C G T A C G A G C T A G T C G C T A A G T C T C A G G A C T A C T G T C G A | E-box(bHLH)/Promoter/Homer | 1e-2 | -6.481e+00 | 0.0054 | 145.0 | 0.65% | 118.7 | 0.45% | motif file (matrix) | svg |
| 104 | T A G C T A G C G A C T C T A G A G C T A G T C G T C A T G C A A C G T A T G C G C T A T G C A | Pbx3(Homeobox)/GM12878-PBX3-ChIP-Seq(GSE32465)/Homer | 1e-2 | -6.425e+00 | 0.0057 | 479.0 | 2.13% | 467.4 | 1.76% | motif file (matrix) | svg |
| 105 | G A C T G C A T C T A G C G A T G A T C T C G A C A T G G A T C | Tgif1(Homeobox)/mES-Tgif1-ChIP-Seq(GSE55404)/Homer | 1e-2 | -6.332e+00 | 0.0062 | 7108.0 | 31.62% | 8069.3 | 30.40% | motif file (matrix) | svg |
| 106 | C G A T T G C A G T A C C G T A A G T C C T A G G A C T C A T G | NPAS(bHLH)/Liver-NPAS-ChIP-Seq(GSE39860)/Homer | 1e-2 | -6.297e+00 | 0.0063 | 3498.0 | 15.56% | 3880.7 | 14.62% | motif file (matrix) | svg |
| 107 | T A C G T A C G G T A C A T C G A C T G T A C G T C G A C T G A T C G A A T C G | E2F6(E2F)/Hela-E2F6-ChIP-Seq(GSE31477)/Homer | 1e-2 | -6.209e+00 | 0.0068 | 840.0 | 3.74% | 864.5 | 3.26% | motif file (matrix) | svg |
| 108 | T G A C C T A G A C T G T A G C C G A T A C T G A T G C C A T G A T C G A T C G A T C G T A G C C T G A T A G C G C T A A C T G C G T A A G C T C G T A C T G A | GATA:SCL(Zf,bHLH)/Ter119-SCL-ChIP-Seq(GSE18720)/Homer | 1e-2 | -6.046e+00 | 0.0080 | 288.0 | 1.28% | 267.9 | 1.01% | motif file (matrix) | svg |
| 109 | T A C G T C G A T A G C A G T C C G T A A G T C C T A G G C A T A C T G A T C G | n-Myc(bHLH)/mES-nMyc-ChIP-Seq(GSE11431)/Homer | 1e-2 | -5.696e+00 | 0.0112 | 1279.0 | 5.69% | 1362.1 | 5.13% | motif file (matrix) | svg |
| 110 | C T A G G C A T G A T C C G T A A G T C T C A G G A C T C T A G | CLOCK(bHLH)/Liver-Clock-ChIP-Seq(GSE39860)/Homer | 1e-2 | -5.579e+00 | 0.0125 | 991.0 | 4.41% | 1041.5 | 3.92% | motif file (matrix) | svg |
| 111 | C T A G A T G C A T G C C G A T A C T G G A C T A T G C G C T A T G A C A G C T T A G C G C T A | PBX1(Homeobox)/MCF7-PBX1-ChIP-Seq(GSE28007)/Homer | 1e-2 | -5.250e+00 | 0.0172 | 170.0 | 0.76% | 150.7 | 0.57% | motif file (matrix) | svg |
| 112 | T A G C G T A C A G T C G T A C C G A T A G T C A G T C A G T C A G T C A G T C C G T A G A T C | Zfp281(Zf)/ES-Zfp281-ChIP-Seq(GSE81042)/Homer | 1e-2 | -5.223e+00 | 0.0175 | 365.0 | 1.62% | 356.9 | 1.34% | motif file (matrix) | svg |
| 113 | T C A G A T C G G A C T A C T G G A C T C A G T C T A G C G T A G T A C C G T A C T A G A T C G | Tbx20(T-box)/Heart-Tbx20-ChIP-Seq(GSE29636)/Homer | 1e-2 | -5.168e+00 | 0.0183 | 455.0 | 2.02% | 454.9 | 1.71% | motif file (matrix) | svg |
| 114 | T G C A A T G C A C G T A C G T A C G T A T G C C T A G A C G T A C G T A G C T G A T C A G C T | T1ISRE(IRF)/ThioMac-Ifnb-Expression/Homer | 1e-2 | -5.056e+00 | 0.0204 | 36.0 | 0.16% | 21.6 | 0.08% | motif file (matrix) | svg |
| 115 | G C T A T A G C A G C T A T C G G T C A C G T A G C T A A T G C G A T C C T G A | IRF4(IRF)/GM12878-IRF4-ChIP-Seq(GSE32465)/Homer | 1e-2 | -4.995e+00 | 0.0214 | 1028.0 | 4.57% | 1092.3 | 4.11% | motif file (matrix) | svg |
| 116 | C G T A C G T A A G C T G A C T T G C A G T C A A C G T A G C T C T G A T C A G | Lhx3(Homeobox)/Neuron-Lhx3-ChIP-Seq(GSE31456)/Homer | 1e-2 | -4.909e+00 | 0.0232 | 3609.0 | 16.06% | 4048.2 | 15.25% | motif file (matrix) | svg |
| 117 | T C G A T A G C T G A C C T G A A G T C A C T G G A C T C A T G | c-Myc(bHLH)/LNCAP-cMyc-ChIP-Seq(Unpublished)/Homer | 1e-2 | -4.848e+00 | 0.0244 | 666.0 | 2.96% | 690.5 | 2.60% | motif file (matrix) | svg |
